# Influencing Factors of Behavior for Reducing Exposure to Endocrine Disrupting Chemicals and Demand for Related Education

**DOI:** 10.3390/ejihpe12030021

**Published:** 2022-03-09

**Authors:** Chae-Min Yoon, Hye-Jin Kim

**Affiliations:** Department of Nursing, Changshin University, Changwon 51352, Korea; 325khj@cs.ac.kr

**Keywords:** barriers, benefits, endocrine disruptors, health promotion, knowledge, students

## Abstract

This study investigates the factors influencing university students’ behavior in reducing exposure to endocrine disrupting chemicals (EDCs) and the demand for related education. This study utilized a descriptive survey. Data were collected from 192 students in Busan, South Korea, from 1 September to 31 December 2020 using an online questionnaire. Data were analyzed using descriptive statistics, independent *t*-test, one-way analysis of variance, Pearson’s correlation coefficient test, and multiple regression. A positive correlation was observed between knowledge about EDCs and perceived benefits, perceived barriers, and behavior for reducing exposure to EDCs. Perceived benefits had a negative correlation with perceived barriers. Factors affecting students’ behavior for reducing exposure to EDCs were age, enrollment in a health-related department, regular exercise, medication, and intake of healthy foods. Moreover, participants preferred to receive relevant information through a variety of educational resources and online teaching methods, favoring videos and social media, but not group discussions or individual counseling. They also preferred lecture-type education and the use of pamphlets, fliers, newspapers, and magazines. Thus, reducing EDC exposure implies encouraging regular exercise, appropriate health medications, and healthy food consumption; it is also necessary to make educational content accessible to college students via the Internet and mobile phones. Future studies should analyze the effect of reducing EDCs within the body through individual behaviors, to improve people’s physical, emotional, and socio-psychological health status.

## 1. Introduction

Endocrine disrupting chemicals (EDCs), more commonly known as environmental hormones, are mostly generated from chemical substances [1]. They have a systemic impact on the body, including the metabolic, nervous, immune, and reproductive systems, as they are absorbed into the body through respiration, food, and skin [2,3]. In recent years, there has been mounting societal interest and concern regarding the health problems posed by EDCs, such as those in antibacterial products for humidifiers, disposable sanitary pads, and particulate matter containing microplastics [4].

In many countries, although relevant agencies such as the Ministry of Environment provide information about EDCs on their websites, this available information is limited; most people state that they obtain information about EDCs through news coverage of EDC-related incidents [5]. However, due to the lack of detailed information promoting behaviors to reduce exposure to EDCs, people are left exposed to these harmful substances without adequate protection. 

There are a variety of theories that attempt to explain behavioral choices to improve one’s health, including the health belief model, pantheory model, rational behavior theory model, and health promotion model [6]. Pender’s health promotion model, particularly, has the advantage of revealing the complex physical, psychological, and social processes that motivate individuals to act toward improving their health [7]. Therefore, this study used Pender’s health promotion model as its theoretical framework.

According to Pender, human behavior depends on the cognition associated with the behavior. Under this framework, perceived benefit, perceived disability, and self-efficacy affect behavioral outcomes [7,8]. Previous studies find that participants’ knowledge about EDCs, perceived disability, and self-efficacy influence their behavior in reducing EDC exposure [4,9,10,11]. One study that investigated health-promoting lifestyles in university students reported that perceived benefits were significantly positively correlated with healthy behaviors [12]. Thus, it is necessary to confirm whether perceived benefits are similarly positively correlated with behaviors that reduce EDCs exposure in college students. The period of college students is an important period that determines the health status of adulthood [13]. EDCs also affect the health of the next generation, namely, the second generation in the long term [14].

Knowledge is an important cognitive factor that alters behavior [15] and has a strong impact on behavioral changes in individuals [16]. A prior study on EDCs in pregnant women reported that behaviors for reducing exposure to EDCs were significantly positively correlated with knowledge about EDCs [14], but a study on university students did not observe a significant correlation between knowledge and behavior [13]. Thus, although both studies were conducted in relation to behaviors for reducing exposure to EDCs, the influencing factors differed depending on the target population. Therefore, further studies to investigate whether knowledge about EDCs predicts behaviors for reducing exposure to EDCs in university students are needed.

In addition, although perceived barriers have been identified as predictors of behaviors for reducing exposure to EDCs [9,10], there are not many studies on EDCs in the context of college students, owing to the low interest in this subject among students.

While studies have reported that there is a great need for education on EDCs [17,18], no previous study has surveyed the need for educating university students in this regard. Moreover, as a healthy lifestyle during the university phase has an important impact on health in adulthood [19], it is important to actively promote a healthy lifestyle among students through EDC-related disease prevention and health management interventions.

In this context, this study aimed to identify the predictors of behaviors for reducing exposure to EDCs in university students, surveying the need to educate them, and presenting foundational data for developing programs that promote such behaviors.

Our study investigated the following relationships to analyze the factors affecting behaviors for reducing exposure to EDCs in university students, based on Pender’s health promotion model:

1. The association between participants’ general and EDC-related characteristics.
2. The differences in behaviors for reducing exposure to EDCs based on the participants’ general and EDC-related characteristics.
3. The participants’ level of knowledge about EDCs, perceived benefits, perceived barriers, and behaviors for reducing exposure to EDCs.
4. The correlations between knowledge about EDCs, perceived benefits, perceived barriers, and behaviors for reducing exposure to EDCs.
5. The predictors of behaviors for reducing exposure to EDCs.
6. The participants’ educational needs pertaining to EDCs.

## 2. Materials and Methods

This study was approved by the Institutional Review Board of Kyungsung University Ethics Committee (KSU-20-04-002-0826). Informed consent was obtained from all the participants.

### 2.1. Design and Participants

We analyzed the predictors of behaviors for reducing exposure to EDCs and relevant educational needs among university students. The sample size was determined using the G*power 3.1 program. For a multiple regression with a medium effect size (f) of 0.15, power (1-*β*) of 0.90, and significance (⍺) of 0.05, the minimum sample size was calculated to be 175; we, therefore, distributed 194 questionnaires, considering a dropout rate of 10%. The dropout rate was low because the participants were recruited using snowball sampling. They were selected from two universities in Busan, South Korea.

The sample consisted only of adults (men: *n* = 53; women: *n* = 139). We removed cases that involved erroneous data entry (*n* = 2), limiting the analytical sample to 192 volunteers, with a mean age of 21.61 ± 2.0 years. According to the age criteria in Daniel Levinson’s theory [20], the study participants were divided into two groups: early adulthood transition (Korean age: 21–24 years, *n* = 177) and early adulthood stage (Korean age: 25–29 years, *n* = 15). Since there are subtle differences between transition and adaptation in early adulthood, the researchers divided participants into these two groups to identify age-based differences.

There were 137 (71.4%) students enrolled in health-related departments, and 55 (28.6%) in other departments. We asked participants about the diagnosis of disease among family members, their perceived current health status, whether they exercised regularly, whether they were taking medication, and whether they were consuming healthy food, among other aspects, to understand their general characteristics.

The inclusion criteria for study participants included those who agreed to participate in the study and university students living in the Busan area. Exclusion criteria were those who did not agree to participate and did not complete the questionnaire.

### 2.2. Assessment Tools

#### 2.2.1. General and EDC-Related Characteristics

General characteristics included age, sex, health-related major, family medical history, current health status, regular exercise, use of drugs for treatment of a disease, and use of health supplements. EDC-related characteristics included awareness of EDCs, prior education about EDCs, and belief regarding the need for education about EDCs; 11 items were used to assess these characteristics.

#### 2.2.2. Knowledge about EDCs

In this study, knowledge about EDCs was measured using the instrument developed by Kim and Kim [21] and further modified by Kim and Park [22]. The correct answer was given 1 point, and wrong answers and “I don’t know” responses were scored 0. A higher score indicates greater knowledge about EDCs. The reliability (Cronbach’s α) was 0.74 at the time of development, 0.84 in the study by Kim and Park [22], and 0.81 in this study.

#### 2.2.3. Perceived Benefit

Perceived benefits were measured using the 11-item Perceived Benefits Scale developed by Moon [23]. Each item is rated on a five-point Likert scale, and the total score ranges from 11 to 55, with a higher score indicating a higher perceived benefit. The reliability (Cronbach’s α) was 0.71 at the time of development, and 0.90 in this study.

#### 2.2.4. Perceived Barrier

Perceived barriers were measured using the six-item Perceived Barriers Scale developed by Kim et al. [24], adapted by Shin and Kang [25], and modified as items for perceived barriers regarding behaviors for reducing exposure to EDCs by Jeon and Um [14]. Each item is rated on a five-point Likert scale, and the total score ranges from 6 to 30, with a higher score indicating a higher perceived barrier. The reliability (Cronbach’s α) was 0.79 at the time of development, 0.84 in the study by Jeon and Um [14], and 0.83 in this study.

#### 2.2.5. Behaviors for Reducing Exposure to EDCs

Behaviors for reducing exposure to EDCs were measured using the instrument developed by Kim and Kim [21] and modified by Kim and Choi [26], which was further validated through evaluation by one nursing and one environmental health professor. This instrument has 35 items, and each item is rated on a five-point Likert scale. The total score ranges from 35 to 175, with a lower score indicating more frequent engagement in behaviors to reduce exposure to EDCs. The reliability (Cronbach’s α) was 0.83 at the time of development and 0.76 in this study.

#### 2.2.6. EDC-Related Educational Needs

EDC-related educational needs were measured using four items: EDC content, method of education, total number of education sessions, and weekly frequency of education sessions.

### 2.3. Statistical Analyses

All analyses were performed using the SPSS statistical package (version 22) at a significance level of 0.05 [27]. This study employed parametric statistics since the data satisfied the condition of normal distribution. Referring to Kim and Kim [21], participants’ general characteristics, EDC-related characteristics, knowledge about EDCs, perceived benefits, perceived barriers, behaviors for reducing exposure to EDCs, and EDC-related educational needs were analyzed using descriptive statistics—namely, frequency, percentage, mean, and standard deviation. The differences in behaviors to reduce exposure to EDCs according to the participants’ general characteristics were analyzed using an independent *t*-test and one-way analysis of variance (ANOVA), with the Scheffé test performed post-hoc. The correlations between knowledge about EDCs, perceived benefits, perceived barriers, and behaviors for reducing exposure to EDCs were analyzed using Pearson’s correlation coefficients. The predictors of behaviors for reducing exposure to EDCs were analyzed using multiple regression analysis. Statistical significance was set at *p* < 0.05.

## 3. Results

### 3.1. Participants’ General and EDC-Related Characteristics

The mean age of the sample was 21 years, and most of the participants (92.2%) were aged between 20 and 24 years. The majority of the participants (72.4%) were women, and 71.4% were health-related majors. A total of 52.6% of the participants had a family member diagnosed with a medical illness, including hypertension or diabetes mellitus, and 55.7% perceived themselves to be healthy. Among the participants, 58.3% did not exercise regularly, and 92.7% did not take drugs to treat an illness. Further, 50.0% said that they were currently taking health supplements. Regarding EDC-related characteristics, 56.8% were not aware of EDCs. The majority (72.9%) had no prior EDC-related education, and 94.8% stated that they would appreciate education about EDCs (Table 1).

### 3.2. Differences in Behaviors for Reducing Exposure to EDCs According to General Characteristics and EDC-Related Characteristics

Participants aged 20–24 years engaged in behaviors for reducing exposure to EDCs significantly more frequently than did those aged 25–29 years (t = −2.047, *p* = 0.042, Cohen’s d = 0.57). Furthermore, the frequency of behaviors for reducing exposure to EDCs was significantly higher among participants with health-related majors (t = −2.193, *p* = 0.030), those who exercised regularly (t = −3.103, *p* = 0.002), those not taking drugs (t = 2.166, *p* = 0.032), and those taking health supplements (t = −2.159, *p* = 0.032). There was no statistically significant difference in exposure perception, educational experience, and related education necessity based on the characteristics linked to EDCs (Table 1).

### 3.3. Knowledge about EDCs, Perceived Benefits, Perceived Barriers, and Behaviors for Reducing Exposure to EDCs

The mean knowledge about EDCs score was 15.71 ± 4.56 out of 24, and the mean perceived benefits score was 41.74 ± 7.08 out of 55. The mean perceived barriers score was 13.99 ± 4.13 out of 30, and the mean score for behaviors for reducing exposure to EDCs was 98.34 ± 13.09 out of 175.

### 3.4. Correlations between Knowledge about EDCs, Perceived Benefits, Perceived Barriers, and Behaviors for Reducing Exposure to EDCs

There was a significant positive correlation between knowledge about EDCs and perceived benefits (r = 0.342, *p* < 0.001), a significant negative correlation between perceived barriers and perceived benefits (r = −0.221, *p* = 0.002), and a significant positive correlation between behaviors for reducing exposure to EDCs and perceived barriers (r = 0.327, *p* < 0.001) (Table 2).

### 3.5. Predictors of Behaviors for Reducing Exposure to EDCs

The frequency of behaviors for reducing exposure to EDCs was higher among those of younger age (β = 0.685, *p* = 0.008), those with health-related majors (β = 0.143, *p* = 0.032), those who exercised regularly (β = 0.218, *p* = 0.001), those who took health supplements (β = 0.200, *p* = 0.003), and those with lower levels of perceived barriers (β = 0.233, *p* = 0.001). These factors explained 20.3% of the variance (F = 9.100, *p* < 0.001). The Durbin–Watson statistic was 1.772, confirming the absence of autocorrelation among residuals; multicollinearity was also confirmed to be absent, with tolerance ranging from 0.923 to 0.955, and the variance inflation factor ranging from 1.047 to 1.083 (Table 3).

### 3.6. Content, Methods, and Frequency of EDC-Related Education

The participants stated that education about EDCs should include the definition, types, features, mechanisms, impact, current management, relevant products, and adverse effects of EDCs, as well as ways to reduce exposure (Table 4).

Furthermore, the participants preferred videos and social media as the channels of education, not favoring either group discussions or individual counseling. They also preferred lecture-type education and the use of pamphlets, fliers, newspapers, or magazines (Table 5). Most of the participants (52.6%) preferred 3–4 sessions of education, followed by 1–2 sessions (40.1%), 5–6 sessions (4.7%), and 7–8 sessions (2.6%). The majority (75.4%) preferred one educational session per week, followed by two sessions per week (18.2%) and three sessions per week (6.4%).

## 4. Discussion

The results of this study revealed that the frequency of behaviors for reducing exposure to EDCs was higher among those of younger age, health-related majors, those who exercised regularly, did not take drugs, and took health supplements.

There was a large significant difference in behavior between the two groups according to age. The 21–24 age group exhibited more reduction behaviors regarding EDC exposure than the 25–29 group did. In the case of Korea, this finding is interpreted as the indirect outcome of classes related to the environment that are conducted as part of the regular syllabus up to high school. Currently, environmental education is not mandatory in the Korean university curriculum. For this reason, the level of interest or understanding regarding EDC exposure reduction is low. Therefore, there is a need for educational programs that can effectively encourage university students to reduce exposure to EDCs.

The mean score for knowledge about EDCs was 15.67 out of 24, which was similar to the scores reported among nursing students [28], pregnant women [9], and mothers of infants and toddlers [22] using the same instrument. As EDCs are known to have the greatest impact on fetuses and in infancy and early childhood [29,30,31], we anticipated that mothers of children in these age groups would have a greater level of knowledge compared to university students, but there were no differences in the level of knowledge between the two groups. Further, we anticipated that nursing students would have a greater level of knowledge as they had more EDC-related education, but their level of knowledge did not differ from that of university students. These results seem to be attributable to the lack of opportunities for education about EDCs or lack of educational programs that provide accurate information about the same among various groups of individuals. Thus, we suggest subsequent studies must develop education programs for EDCs that are tailored to different target groups.

The mean perceived benefits score was 41.63 out of 55, which was similar to that reported for single women using the same instrument [26]. Our results were consistent with previous findings showing a significant positive correlation between health promoting behaviors and perceived benefits in university students; however, it is difficult to compare our findings with prior literature due to the lack of other studies analyzing the relationship between behaviors for reducing exposure to EDCs and perceived benefits. Therefore, further studies are needed to examine the relationship between perceived benefits and behaviors for reducing exposure to EDCs.

The mean perceived barrier score was 14.14 out of 30, which was similar to the score among single women measured using the same instrument [26]. This was also consistent with the scores reported for breastfeeding and pregnant women [9,10], although these studies used a different instrument. These results show that a lower perceived barrier has a positive impact on health promoting behaviors. However, a prior study that analyzed the relationship between perceived barriers and health promoting behaviors in university students [32] did not observe a significant relationship between the two, contradicting our results. This suggests that being inundated with too much relevant information increases individuals’ perceived barriers. Hence, education programs that promote behaviors for reducing exposure to EDCs should not include too much information, to lower participants’ perceived barriers.

Regarding the scale for behaviors for reducing exposure to EDCs, a lower score indicates more frequent engagement in behaviors to reduce exposure to EDCs. In our study, the total score among university students was 98.3 out of 175, which would translate to 56.2 from 100 points. This is slightly below the score of 60.6 reported among single women in a previous study [28], which contradicts our hypothesis that single women would be more interested in the topic. The finding that the two groups showed a difference in the frequency of behaviors for reducing exposure to EDCs despite no difference in the level of knowledge about EDCs may be attributable to the fact that there is a growing interest in environmental issues in recent years—particularly the problem of particulate matter in sanitary pads—with individuals having more exposure to relevant information owing to more frequent media coverage of issues pertinent to behaviors for reducing exposure to EDCs. If a simple exposure to relevant issues and media coverage could promote behaviors for reducing exposure to EDCs, more specialized education is expected to produce better outcomes. Thus, we suggest intervention studies to develop and evaluate the effects of programs that promote behaviors for reducing exposure to EDCs, by accurately delivering specialized and useful information, thereby motivating actual practice.

In contrast to our expectation that knowledge about EDCs and perceived benefits would be the most potent predictors of behaviors for reducing exposure to EDCs, a regression analysis revealed that age, regular exercise, use of health supplements, and perceived barriers were the main predictors of behaviors for reducing exposure to EDCs, explaining 20.3% of the variance in scores. These results are consistent with previous studies [14] that investigated factors related to EDCs in the same study population. However, these results do not reflect the findings of previous studies that showed a positive correlation with the perceived benefits of college students’ healthful behaviors [12]. Nonetheless, the results are in line with the results of a study on stress and health in nursing students, who found that maintaining a good lifestyle, such as regular exercise and healthy food intake, has a positive effect on health [33]. In addition, in a study [34] investigating lifestyles of adolescents with headaches, inappropriate dietary habits such as obesity, caffeine intake, and eating habits were identified as factors causing pain.

These results show that although providing accurate knowledge is important in inducing behavioral changes, eliminating barriers is more effective in promoting healthful behaviors. Thus, we suggest curricula that include content that can increase knowledge about EDCs, thereby lowering the barriers, when developing EDC-related educational programs. The program should include appropriate exercise, medication intake, and healthy food choices.

Regarding the need for EDC-related educational content, the participants preferred to learn about the definition, types, features, impact, relevant products, and adverse effects of EDCs, including specific practices for reducing exposure, compared to learning about the mechanisms of EDCs and current management trends. This suggests that individuals wish to learn more about practical aspects, such as the actual impact and practices of reducing exposure to EDCs, as opposed to an in-depth knowledge about the topic. Regarding the educational methods, the participants preferred videos or social media to lectures, pamphlets, fliers, newspapers, or magazines. This reflects general recent trends in which university students have become more familiar with online education during the COVID-19 pandemic, preferring educational methods without spatial restrictions. Some benefits of online education are that it does not confine the program to a specific setting or number of people; hence, online education would be a useful means for programs that aim at promoting behaviors for reducing exposure to EDCs. Although online education has the advantage of being open to time and place, it has disadvantages compared to face-to-face education in terms of the effectiveness, level of participation, and concentration. Therefore, it is necessary to plan the program taking these negative aspects into account. The participants did not prefer group discussions and individual counseling, which also reflects recent trends in preferences regarding means of education among university students. However, continued motivation and management are essential to promote a steady engagement in health promoting behaviors. Therefore, measures to continuously manage these behaviors while considering participants’ needs should be explored. Future studies should develop and analyze the effects of relevant programs.

This study has a few limitations. First, as this study was conducted in a single location, the results may not apply to university students in different settings. In addition, there are limitations in generalizing the research results because economic and demographic aspects, including the physical environment of the selected research participants, were not considered. Second, since this study was cross-sectional in nature, it could not determine the temporal relationship between the variables. Third, due to the coronavirus pandemic situation, the study schedules ended earlier than planned, negatively affecting the sample size. Future studies with a larger sample size are recommended to provide more information.

In addition, we suggest that future studies should analyze the effect of reducing the amount of EDCs (e.g., bisphenol A, phthalate, etc.) within the body through behaviors for reducing exposure to EDCs, thus improving the physical, emotional, and socio-psychological health status of individuals.

## 5. Conclusions

Our study has important implications because we identified the key factors influencing the level of knowledge of EDCs among individuals in their twenties residing in Korea, as well as their behaviors for reducing exposure to EDCs. As confirmed in this study, in order to reduce EDC exposure, regular exercise should be strengthened, appropriate health medications should be taken, and healthy foods should be consumed. In addition, it is necessary to provide college students with a content delivery program to increase perceived disability. In order to reduce exposure to EDCs among college students, education should be readily accessible at any time via mobile phones or the Internet.

## Figures and Tables

**Table 1:** Differences in behaviors for reducing exposure to EDCs based on participants’ general and EDC-related characteristics (*n* = 192).

Characteristics		Categories	*n* (%) or *M ± SD*	Behavior for Reducing Exposure to EDCs	t (F)	*p*
				*M* ± *SD*		
General characteristics						
	Age (years)		21.61 ± 2.0		−2.047	0.042
		20−24	177 (92.2)	97.78 ± 13.09		
		25−29	15 (7.8)	104.93 ± 11.58		
	Gender	Male	53 (27.6)	97.75 ± 12.23	−0.384	0.701
		Female	139 (72.4)	98.57 ± 13.44		
	Health-related department enrollment	Yes	137 (71.4)	97.04 ± 13.48	−2.193	0.030
		No	55 (28.6)	101.58 ± 11.57		
	Diagnosis of disease among family members	Yes	101 (52.6)	98.89 ± 12.02	0.609	0.543
		No	91 (47.4)	97.74 ± 14.23		
	Perceived current health status	Healthy	77 (40.1)	98.34 ± 13.09	0.404	0.668
		Usual	107 (55.7)	98.05 ± 12.97		
		Unhealthy	8 (4.2)	102.38 ± 15.86		
	Regular exercise	Yes	80 (41.7)	94.95 ± 13.05	−3.103	0.002
		No	112 (58.3)	100.76 ± 12.63		
	Medication	Yes	14 (7.3)	105.57 ± 13.84	2.166	0.032
		No	178 (92.7)	97.78 ± 12.90		
	Taking health supplements	Yes	96 (50.0)	96.32 ± 13.29	−2.159	0.032
		No	96 (50.0)	100.36 ± 12.64		
EDC-related characteristics						
	Recognition of exposure to EDCs	Yes	83 (43.2)	97.28 ± 12.34	−0.985	0.326
		No	109 (56.8)	99.16 ± 13.64		
	EDC-related educational experience	Yes	52 (27.1)	97.82 ± 13.34	−0.333	0.740
		No	140 (72.9)	98.54 ± 13.04		
	Recognizing the need for education related to EDCs	Yes	182 (94.8)	98.32 ± 13.39	−0.132	0.897
		No	10 (5.2)	98.60 ± 5.68		

Abbreviations: EDCs, endocrine disrupting chemicals; *M*, mean; *SD*, Standard deviation.

**Table 2:** Correlations between knowledge about EDCs, perceived benefits, perceived barriers, and behaviors for reducing exposure to EDCs (*n* = 192).

Variable	Knowledge about EDCs	Perceived Benefits	Perceived Barriers	Behavior for Reducing Exposure to EDCs
Knowledge about EDCs				
Perceived benefits	0.34 **			
Perceived barriers	−0.02	−0.22 *		
Behavior for reducing exposure to EDCs	0.00	−0.03	0.32 **	

* *p* < 0.01. ** *p* < 0.001.

**Table 3:** Regression results for factors influencing the behaviors for reducing exposure to EDCs (*n* = 192).

	B	S.E	β	t (*p*)	R^2^	Adjusted R^2^	F (*p*)
(Constant)	71.677	9.020		7.947 (<0.001)	0.228	0.203	9.100 (<0.001)
Age (years)	4.728	1.761	0.178	2.685 (0.032)			
Health-related department enrollment	4.138	1.918	0.143	2.158 (0.686)			
Regular exercise	5.783	1.751	0.218	3.302 (0.001)			
Medication	−6.576	3.377	−0.131	−1.947 (0.053)			
Taking health supplements	5.231	1.743	0.200	3.001 (0.003)			
Perceived barriers	0.741	0.213	0.233	3.481 (0.001)			

**Table 4:** Demand for educational content (*n* = 192).

Contents of Program	Not Very Necessary *n* (%)	Unnecessary *n* (%)	Neutral *n* (%)	Necessary *n* (%)	Very Necessary *n* (%)
Definition of EDCs	2 (1.0)	4 (2.1)	42 (21.9)	96 (50/0)	48 (25)
Types of EDCs	-	3 (1.6)	25 (13.0)	103 (53.6)	61 (31.8)
Features of EDCs	-	4 (2.1)	34 (17.7)	96 (50.0)	58 (30.2)
Mechanisms of EDCs	2 (1.0)	25 (13.0)	57 (29.7)	70 (36.5)	38 (19.8)
Effects of EDCs	-	3 (1.6)	18 (9.4)	82 (42.7)	89 (46.4)
Management status of EDCs	2 (1.0)	9 (4.4)	54 (28.1)	78 (40.6)	49 (25.5)
Products containing EDCs	-	3 (1.6)	10 (5.2)	76 (39.6)	103 (53.6)
Side effects of EDCs	-	5 (2.6)	14 (7.3)	87 (45.3)	86 (44.8)
How to reduce exposure to EDCs	-	3 (1.6)	11 (5.7)	94 (43.8)	94 (49.0)

**Table 5:** Demand regarding educational methods (*n* = 192).

Methods	Not Very Appropriate *n* (%)	Not Appropriate *n* (%)	Neutral *n* (%)	Appropriate *n* (%)	Very Appropriate *n* (%)
Lecture	4 (2.1)	7 (3.6)	54 (28.1)	100 (52.1)	27 (14.1)
Video	-	3 (1.6)	33 (17.2)	1114 (59.4)	42 (21.9)
PPT and print etc.	9 (4.7)	7 (3.6)	56 (29.2)	86 (44.8)	34 (17.7)
Books and newspapers, etc.	10 (4.7)	26 (13.5)	78 (40.6)	58 (30.2)	21 (10.9)
Group discussion	33 (17.2)	58 (30.2)	69 (35.9)	24 (12.5)	8 (4.2)
Individual counseling	34 (17.7)	46 (24.0)	71 (37.0)	25 (13.0)	16 (8.3)
SMS	4 (2.1)	11 (5.7)	38 (19.8)	79 (41.1)	60 (31.3)

Abbreviations: PPT, Power Point; SMS, short message service.

## Data Availability

Not Applicable.
